# A New Pathogen Transmission Mechanism in the Ocean: The Case of Sea Otter Exposure to the Land-Parasite *Toxoplasma gondii*

**DOI:** 10.1371/journal.pone.0082477

**Published:** 2013-12-18

**Authors:** Fernanda F. M. Mazzillo, Karen Shapiro, Mary W. Silver

**Affiliations:** 1 Department of Ocean Sciences, University of California Santa Cruz, Santa Cruz, California, United States of America; 2 Department of Pathology, Microbiology and Immunology, School of Veterinary Medicine, University of California, Davis, California, United States of America; University at Buffalo, United States of America

## Abstract

*Toxoplasma gondii* is a land-derived parasite that infects humans and marine mammals. Infections are a significant cause of mortality for endangered southern sea otters (*Enhydra lutris nereis*), but the transmission mechanism is poorly understood. Otter exposure to *T. gondii* has been linked to the consumption of marine turban snails in kelp (*Macrocystis pyrifera*) forests. It is unknown how turban snails acquire oocysts, as snails scrape food particles attached to surfaces, whereas *T. gondii* oocysts enter kelp beds as suspended particles via runoff. We hypothesized that waterborne *T. gondii* oocysts attach to kelp surfaces when encountering exopolymer substances (EPS) forming the sticky matrix of biofilms on kelp, and thus become available to snails. Results of a dietary composition analysis of field-collected snails and of kelp biofilm indicate that snails graze the dense kelp-biofilm assemblage composed of pennate diatoms and bacteria inserted within the EPS gel-like matrix. To test whether oocysts attach to kelp blades via EPS, we designed a laboratory experiment simulating the kelp forest canopy in tanks spiked with *T. gondii* surrogate microspheres and controlled for EPS and transparent exopolymer particles (TEP - the particulate form of EPS). On average, 19% and 31% of surrogates were detected attached to kelp surfaces covered with EPS in unfiltered and filtered seawater treatments, respectively. The presence of TEP in the seawater did not increase surrogate attachment. These findings support a novel transport mechanism of *T. gondii* oocysts: as oocysts enter the kelp forest canopy, a portion adheres to the sticky kelp biofilms. Snails grazing this biofilm encounter oocysts as ‘bycatch’ and thereby deliver the parasite to sea otters that prey upon snails. This novel mechanism can have health implications beyond *T. gondii* and otters, as a similar route of pathogen transmission may be implicated with other waterborne pathogens to marine wildlife and humans consuming biofilm-feeding invertebrates.

## Introduction

*Toxoplasma gondii* is a coccidian parasite that infects humans and warm-blooded animals [1]. Infections with this terrestrial parasite have also been documented in marine mammals [2], including the southern sea otter (*Enhydra lutris neries*). The southern sea otter is an endangered subspecies that inhabits giant kelp forests, including those of *Macrocystis pyrifera,* along the California coast. *Toxoplasma gondii* is recognized as a significant cause of mortality in southern sea otters, with harmful consequences for the health and recovery of this population [3]. Infected otters have been detected at several locations along the coast of California, and high-risk sites for otter exposure were described in populations from Morro Bay and Cambria, California [4], [5].

The transmission mechanism of *T. gondii* in the marine food web is unknown. Sea otter exposure to this parasite is thought to occur through ingestion of oocysts that are shed by felids [6], [7]. Cats can shed hundreds of millions of oocysts in their feces when infected, and only 1–10 oocysts are needed to successfully infect mammals [8], [9], [10]. *Toxoplasma gondii* oocysts may reach coastal waters via contaminated freshwater runoff [4], and wetland degradation may increase flux of oocysts by more than 2 orders of magnitude [11]. Oocysts introduced into coastal waters may survive for at least 24 months [12], and may adhere to aquatic aggregates or occur freely in the water column [13].

Marine invertebrates (e.g. mussels and oysters) and vertebrates (e.g. anchovies and sardines) that feed by filtering seawater through their gills may acquire *T. gondii* oocysts from seawater [14]–[17]. These animals may then serve as potential sources of infection of *T. gondii* to marine mammals. However, southern sea otters are diet specialists and a recent epidemiological study showed that otters that specialize on marine turban snails (*Chlorostoma brunnea, C. montereyi, and Promartynia pulligo –* formally assigned to the genus *Tegula*) in the kelp forest are 12 times more likely to acquire *T. gondii* than those consuming other prey [5]. As opposed to mussels, oysters, sardines and anchovies, which feed by filtering food particles out of the water, turban snails scrape surfaces using a radula to ingest food particles that are attached to a substrate. Thus, the mechanism by which the benthic feeding marine turban snails acquire oocysts suspended in the water is puzzling and suggests an alternate mode of feeding on small planktonic particles.

Because of their feeding strategy, turban snails are likely to consume biofilm-associated organisms that colonize kelp surfaces [18]–[20]. Biofilms are defined as mixed assemblages of microbes enclosed in a matrix, adhering to each other and/or to surfaces [21]. Extracellular polymeric substances (EPS) form the matrix of biofilms and are defined as a complex of high molecular weight macromolecules, mainly polysaccharides, localized outside the cell wall in the form of gels, slime or capsules [22]–[24]. Because of its adhesive properties, EPS is regarded as the ‘biological glue’ that anchors biofilm microorganisms (e.g., bacteria and microalgae) to a surface [25]. Several studies have addressed the role of EPS in the attachment of bacteria and diatoms to surfaces such as macroalgae, ice and sediments [26]–[28]. Microorganisms such as benthic diatoms and fungi have been observed on the surface of *M. pyrifera* blades [20], [29], [30]. These organisms may be trapped in a sticky EPS matrix that possibly helps them attach to the surface of the kelp blades [31]–[33].

A secondary source of mucilage-rich materials that may form biofilms on kelp includes waterborne TEP (transparent exopolymer particles) - a particulate form of EPS in the water column [34]. Major producers of TEP in the water column include bacteria and phytoplankton, and TEP is regarded as a major biofilm agent [35]. When water upstream of a kelp bed contains TEP, the TEP may make contact with kelp surfaces as it flows through the kelp bed, subsequently adhering directly to the kelp or to EPS-coated surfaces of the kelp. A recent study documented this phenomenon, showing colonization of TEP by bacteria and microalgae suspended in the water, and subsequently adhering to glass surfaces rapidly (e.g. 30 min), promoting biofilm development [36].

Biofilms occur on a wide range of surfaces (natural or man-made) and EPS is also a major component of diatom films found on underwater man-made surfaces [37]. The removal of pathogens from water onto biofilms that colonize the surfaces of pipes or other structures of water treatment plants has been reported [38]. Investigating the potential for *T. gondii* to adhere to biofilms is significant not only for understanding infection mechanisms of marine fauna, but also due to numerous reports of waterborne outbreaks of toxoplasmosis in humans worldwide [39]. To date, the adhesion of coccidian parasites' oocysts has not been linked to EPS of biofilms that colonize natural surfaces such as kelp blades.

In this study, our major goals were to determine whether EPS on kelp surfaces and/or TEP in the water promote adhesion of *T. gondii* oocysts to kelp in a central California forest of *Macrocystis pyrifera*. We also investigated, as a secondary goal, the similarity of epiphytic organisms within the kelp blade biofilm to the organisms (or their remains) present within the feces of snails that had been feeding on those same blades. Here we hypothesize that once *T. gondii* are trapped in the kelp biofilm via EPS or TEP, the parasite is available for ingestion by marine turban snails and other benthic-scraping organisms. This hypothesis could help explain why sea otters that have specialized diets on marine turban snails are more likely to be infected with *T. gondii* than those consuming other prey.

## Methods

Two experiments were designed to: (1) provide qualitative data on snail diet and kelp biofilm composition; and (2) test whether *T. gondii* oocyst surrogates adhere to kelp blade surfaces via EPS on the blades and/or via TEP suspended in the water that subsequently become biofilm on the blades. Together these experiments examine the possible mechanism(s) by which *T. gondii* oocysts may become associated with kelp surfaces and whether snails can consume organisms entrained within the kelp biofilm: if such associations are found, then a delivery route of the planktonic oocysts to the endangered otter would be identified for the first time. We considered both EPS (the matrix of the biofilm on kelp surfaces) and TEP in the water, as potential agents that could deliver *Toxoplasma* to snails that graze the kelp surfaces with biofilms. Dragon green microspheres that have been previously validated as surrogate particles for *T. gondii* oocysts were used because they have surface properties (i.e., size, specific gravity, hydrophobicity, and surface charge) that resemble those of *T. gondii* oocysts [40]. Due to the biohazardous nature of *T. gondii* oocysts, employing surrogates in mesocosm experiments provides an alternative approach for evaluating the waterborne transport of this zoonotic pathogen. Previous studies have successfully applied these surrogates to demonstrate waterborne transport of oocysts [11] and their association with macroaggregates [13].

### Experiment 1: Turban snail diet and kelp biofilm composition

On Aug 8 and 24, 2011, 30 sexually mature (shell>1.5 cm) and juvenile (shell <1.5 cm) turban snails (*Chlorostoma* spp.), along with the kelp blades they were associated with, were collected by hand onboard the *R/V Sebastes* from the canopy of kelp beds in Carmel (36°30′57″N and 121°57′18″W), approximately 44 km south of the Santa Cruz site that provided material for Experiment 2. Snails and kelp were collected under the permit ID 12119 issued by California Department of Fish and Game. Snails were transported live to the laboratory and placed in 1 L jars with 0.2 µm filtered seawater and associated kelp blades, with one snail and 1 frond placed in each jar and incubated for 16 hrs. Temperature at 15°C and 12 hrs light cycle were maintained. The snail fecal pellets produced in the jars were then recovered, their associated blade stored for later analysis of its biofilm, and pellets stored for mounting on glass slides. The biofilm present on the kelp blade from each snail-grazing container was removed using a PTFE spatula, while submerged in 0.2 µm filtered seawater. Bacteria were visualized by staining biofilm samples with 40 µL of 4′,6-diamidino-2-phenylindole (DAPI) (Pierce Biotechnology Inc., Rockford, Il, USA) (final concentration of 500 mg mL^−1^). Snail pellet content and biofilm organisms from kelp blades on which they had grazed were examined using a Zeiss Axio Imager with phase contrast and a 50 W light source fitted with 2 DAPI bandpass filter sets (wavelength excitation (λex) 350 nm, (wavelength emission) λem >420 nm and λex 350 nm, λem >460 nm).

To visualize the underlying EPS matrix of the kelp biofilm, cross-sections of the kelp blades were stained using 500 µl of pre-filtered (0.2 µm), 0.02% aqueous alcian blue solution (8GX) in 0.06% of acetic acid (pH = 2.5) (ABS). EPS was visualized under bright field on the microscope described above. All micrographs were obtained using an Axio Cam HRc camera system.

### Experiment 2: *T. gondii* surrogate adhesion to kelp blades via EPS

Seawater and kelp blades used in this laboratory experiment were collected at 0700 on July 11, 2012 from the surface of a kelp bed canopy in Santa Cruz (36°56′57″N 122°02′05″W) on board the *R/V Sebastes*. Six kelp blades of similar size (Mean surface area 242 cm^2^±54) and ridged texture, and free of obvious macroscopic epibionts were collected from the same stipe. Surface seawater from the kelp canopy was collected with a bucket. Samples were transported to the laboratory in a cooler with ice and the experiment was initiated immediately. Artificial kelp blades (BioModels Co., Aguanga, CA 92536) were also used in the experiments, as they had not previously been submerged in seawater, and thus provided a control substrate free of biofilm and EPS.

**Figure 1:**
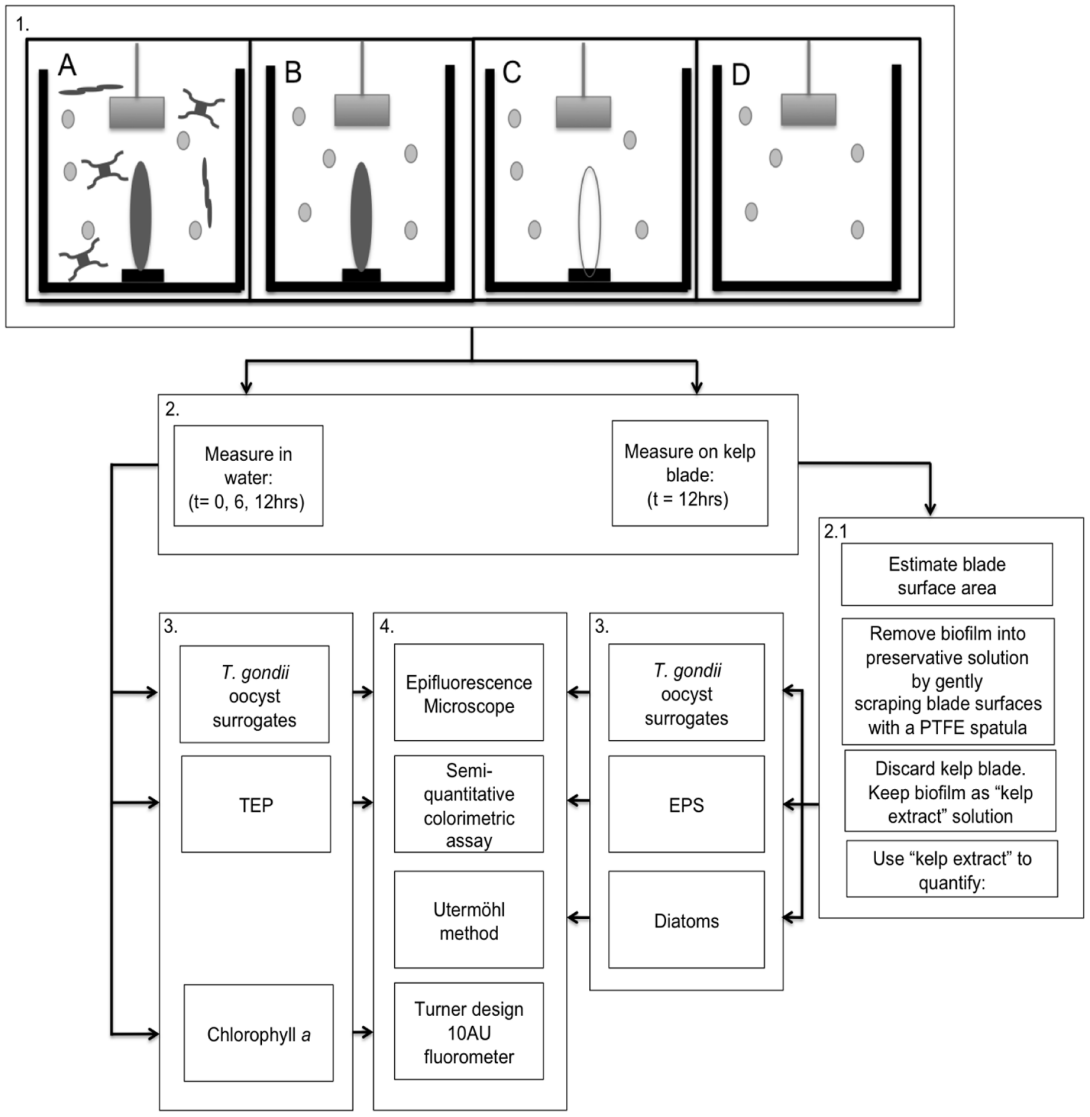
Design of experiment 2. Box 1 shows different treatments A to D, in 4: A) Simulation of kelp forest canopy with seawater and kelp blade; B) Kelp blade and filtered seawater to remove phytoplankton and control for TEP produced in seawater; C) Synthetic kelp blade and filtered seawater to control for EPS on blade and TEP in seawater; D) Filtered seawater to control for surrogate loss due to settling or attachment to surfaces of jar. Box 2 indicates where and when measurements were taken and box 2.1. shows steps to measure parameters on kelp blade biofilm. Box 3 shows parameters measured and box 4 indicates methods used for each parameter.

Four treatments were used to test whether surrogates of *T. gondii* oocysts would become associated with the EPS matrix on the surface of the field-collected kelp blades. Each treatment included 3 replicates in 4 L glass jars. All jars were pre-washed with 10% HCl. Treatment A was designed to reproduce the kelp forest canopy environment and consisted of jars with a kelp blade and unfiltered surface seawater; treatment B consisted of jars with a kelp blade and 0.2 µm filtered seawater. Filtered seawater (0.2 µm) was used to remove any TEP suspended in the water that may have been produced by phytoplankton or bacteria. Treatment C was designed as a control treatment for TEP and EPS by using 0.2 µm filtered seawater and a synthetic kelp blade, and treatment D was designed as a control for the loss of surrogates due to settling or attachment to surfaces of the jar and utilized 0.2 µm filtered seawater without kelp blades (Fig.1).

The 12 hr-controlled experiment was performed on a stir table, with paddles in each treatment jar stirring the water at 30 rpm. Water temperature was held at 12°C, and the experiment was conducted during a light cycle. At the start of the experiment, *T. gondii* surrogate microspheres labeled with a dragon green fluorochrome (Bangs Laboratory, FC07F 5493) were added to each replicate to achieve a final concentration of 20 per mL.

### Quantifying TEP, surrogates, and chlorophyll a in water

Aliquots of 600 mL were collected gently from each treatment at 3 time points (0, 6 and 12 hrs) to quantify the naturally occurring TEP, chlorophyll *a* concentrations, and spiked surrogates in the seawater. Aliquots of 550 mL for TEP and surrogate quantification were preserved with formaldehyde to achieve a 1% final concentration, and then stored at 4°C in the dark for later analysis. TEP was measured by filtering 3 replicates of 100–200 mL aliquots onto 0.4 µm polycarbonate filters. TEP concentrations were determined using a standard semiquantitative colorimetric assay [41].

Surrogates were quantified by filtering 3 replicates of 50 mL onto 0.4 µm polycarbonate filters [42]. Filters were mounted on slides and surrogates were enumerated using a Zeiss Axio Imager fitted with a FITC band pass filter set (λex 460–500 nm, λem 510–560 nm) and a 50 W light source.

Chlorophyll *a* was measured to verify that phytoplankton cells were absent from control treatments B, C and D, but present in treatment A. Two replicates of 25 mL were filtered onto GF/F filters and kept in a −20°C freezer. Chlorophyll *a* was extracted for 24 hrs in 90% acetone and subsequently analyzed on a Turner Design 10AU fluorometer [43].

### Quantifying EPS, surrogates, and the benthic diatom community on kelp blades

At the end of the experiment, each kelp blade was removed from its jar and placed in a preservative solution consisting of 550 mL 0.2 µm filtered seawater solution and formaldehyde at 1% final concentration. Kelp blades were scraped on a glass tray while submerged in its preservative solution. Each kelp blade was photographed with a Nikon digital camera and kelp surface area was measured using the Image J image-analysis software (W.S. Rasband, Image J, U.S. national Institute of Health, http://rsb.info.nih.gov/ij/). The surfaces of kelp blades were scraped on both sides using a spatula to remove the biofilm (i.e., EPS and microorganisms) and *T. gondii* surrogates, taking care not to remove kelp tissue cells. A small PTFE spatula was used to access material on the ridges of the kelp blade. Immediately after scraping was completed, the blade was removed from the tray and the solution with the scraped material from the kelp blade was stored in 1 L glass jars in the dark at 4°C. This solution is subsequently referred to as ‘kelp extract solution’.

To quantify EPS on kelp blades, we adapted the semiquantitative colorimetric assay to quantify TEP. Both TEP and EPS may be measured with this assay [28], [44]. The principle of the method lies in the staining of extracellular polysaccharides with ABS. Alcian blue has been used to stain extracellular polysaccharides in colony matrices or capsules of algae and bacteria [28], [44], [45]. This stain complexes carboxyl (-COO^−^) and half-ester sulfate (OSO_3_^−^) reactive groups of acidic polysaccharides, the main components of EPS, allowing these otherwise transparent substances to be visualized and quantified by measuring its maximum absorbance on a spectrophotometer set at 787 nm [46].

Aliquots of 15 mL from the kelp extract solution (3 replicates per jar) were filtered onto 0.4 µm polycarbonate filters with low, constant vacuum pressure (≤150 mm of Hg). Filters were stained for 2 seconds with 500 µl of ABS, rinsed with distilled water to remove excess dye, and stored in 15 mL centrifuge tubes in a −20°C freezer for up to 2 weeks. Extraction of polysaccharides from filters was done with 5 mL of 80% H_2_SO_4_ for 2 hrs and absorbance of alcian blue was measured on a UV-1201 UV-VIS spectrophotometer set at 787 nm. A calibration curve using gum xantham as the standard was constructed to generate a conversion factor (*F*-factor) to relate the absorbance of stained EPS to the weight of EPS [40]. Final EPS concentration is reported as µg of gum xantham equivalents per cm^2^ of kelp blade surface by adapting the following equation from [41]:

EPS per cm^2^ of kelp blade  =  ((absorbance – blank) ÷ volume filtered)×(kelp extract solution ÷ kelp blade surface area) × *F*-factor

To quantify surrogates on kelp blades and visualize the epiphytic diatoms within the EPS matrix, 3 replicates of 50 mL of the kelp blade extract solution were filtered onto 0.4 µm polycarbonate filters and stained for 2 sec with 500 µl ABS (see above). Filters were mounted on slides and observed with a Zeiss Axio Imager. *Toxoplasma* surrogates were enumerated as mentioned above. EPS was visualized with bright field and epiphytic diatoms with a chlorophyll filter set (λex 440–470 nm, λem >515 nm) and a 50 W light source.

To enumerate diatoms associated with the kelp blade, aliquots of 10 mL of kelp extract solution were settled for 24 hrs in an Utermöhl chamber following the Utermöhl method [47]. A minimum of 100 cells (benthic or planktonic diatoms genera) was counted per chamber using an inverted microscope (Olympus IMT-2).

### Statistical analysis

Biostat 3.0 was used for all statistical comparisons. Mann-Whitney (or U test) was used to test whether the concentration of *T. gondii* oocysts surrogates decreased between two time points (6 hrs and 12 hrs) in all treatments. Mann-Whitney was also used to test whether the percentage of surrogates suspended in water and associate to kelp blades differed at the end of the experiment in each treatment. Kruskal-Wallis (or H test) tested whether the proportion of *T. gondii* oocysts surrogates associated with kelp blades differed among the treatment that mimicked the kelp forest canopy (A), the treatment that controlled for TEP (B) and the control treatment (C).

## Results

### Experiment 1: *Turban snail diet and kelp biofilm composition*

**Figure 2:**
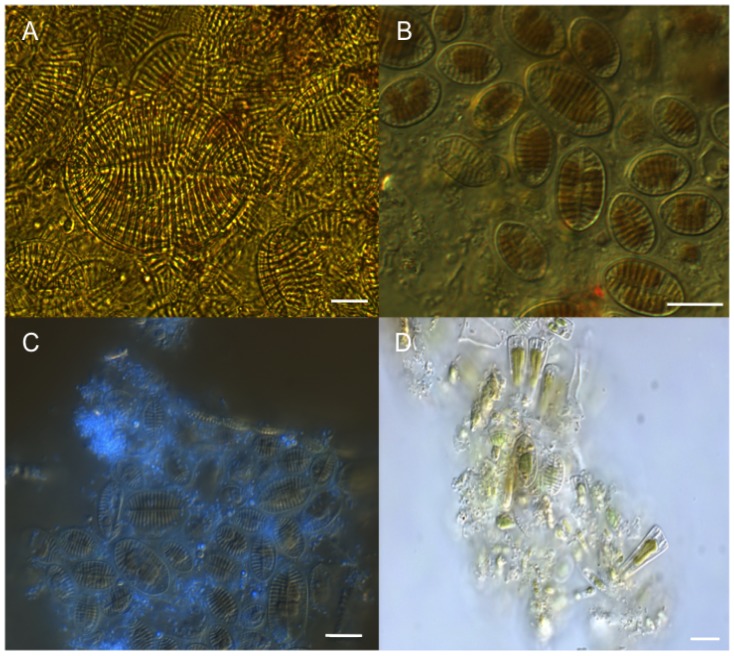
Benthic diatoms (predominantly *Cocconeis* spp.) in snail fecal pellet (A) and in gel-like EPS matrix scraped from surface of giant kelp blades on which snails were feeding (B); DAPI-stained bacteria (C) and different genera of benthic diatoms (D) also in gel-like EPS matrix from kelp blade surfaces from Experiment 1. Scale bars 10 µm.

**Figure 3:**
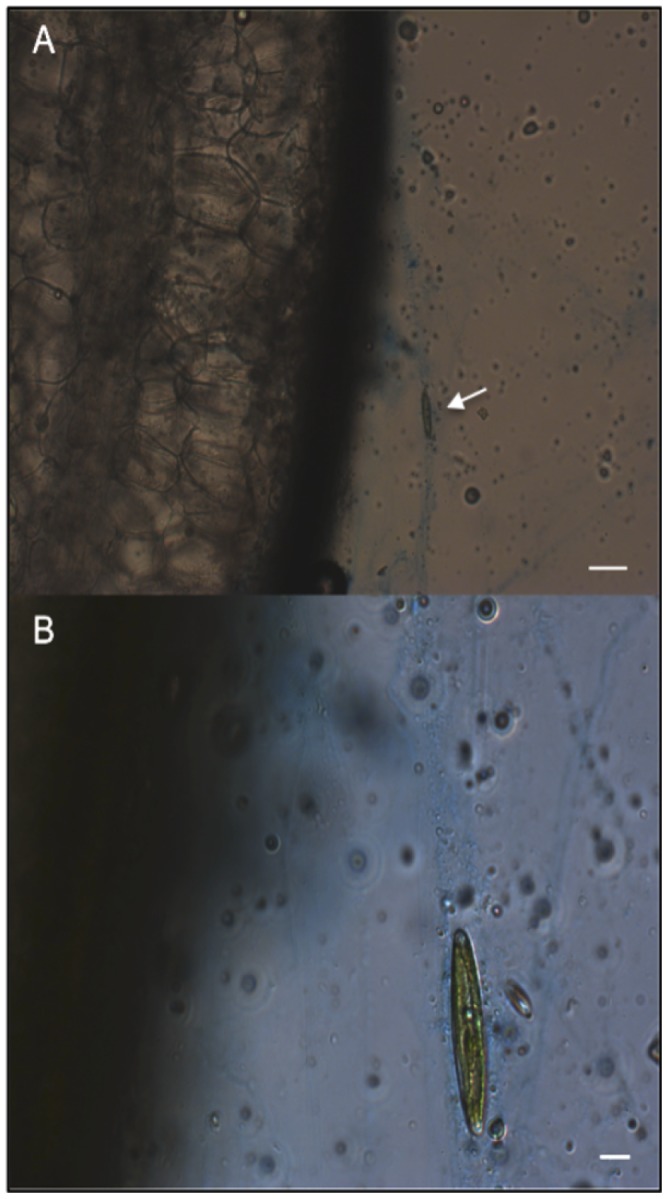
Kelp blade cross-section with diatom (white arrow) ‘trapped’ in EPS fibers, stained with alcian blue (Experiment 1). Scale bar 50 µm. Panel B represents a higher magnification of A, showing benthic diatom in kelp EPS. Scale bar 10 µm.

Turban snail (*Chlorostoma* spp.) fecal pellets produced over the 16-hour grazing incubation with associated kelp blades contained microorganisms similar to those of the biofilm community on the individual blade on which they had grazed. Benthic diatoms resembling *Cocconeis* spp. were dominant in snail pellets (Fig. 2A) and on the surface of kelp blades (Fig. 2B). Bacteria were also observed associated with the *Cocconeis* spp. in the kelp biofilm (Fig. 2C). Other benthic diatoms, including species within the genus *Licmophora*, and possibly *Navicula,* were observed as part of the kelp biofilm (Fig. 2D) and ‘trapped’ in the EPS fibers (Fig. 3A, B). These genera were less abundant relative to *Cocconeis* and not observed in the snail's fecal pellets.

### Experiment 2: *T. gondii surrogate adhesion to kelp blades via EPS*

**Figure 4:**
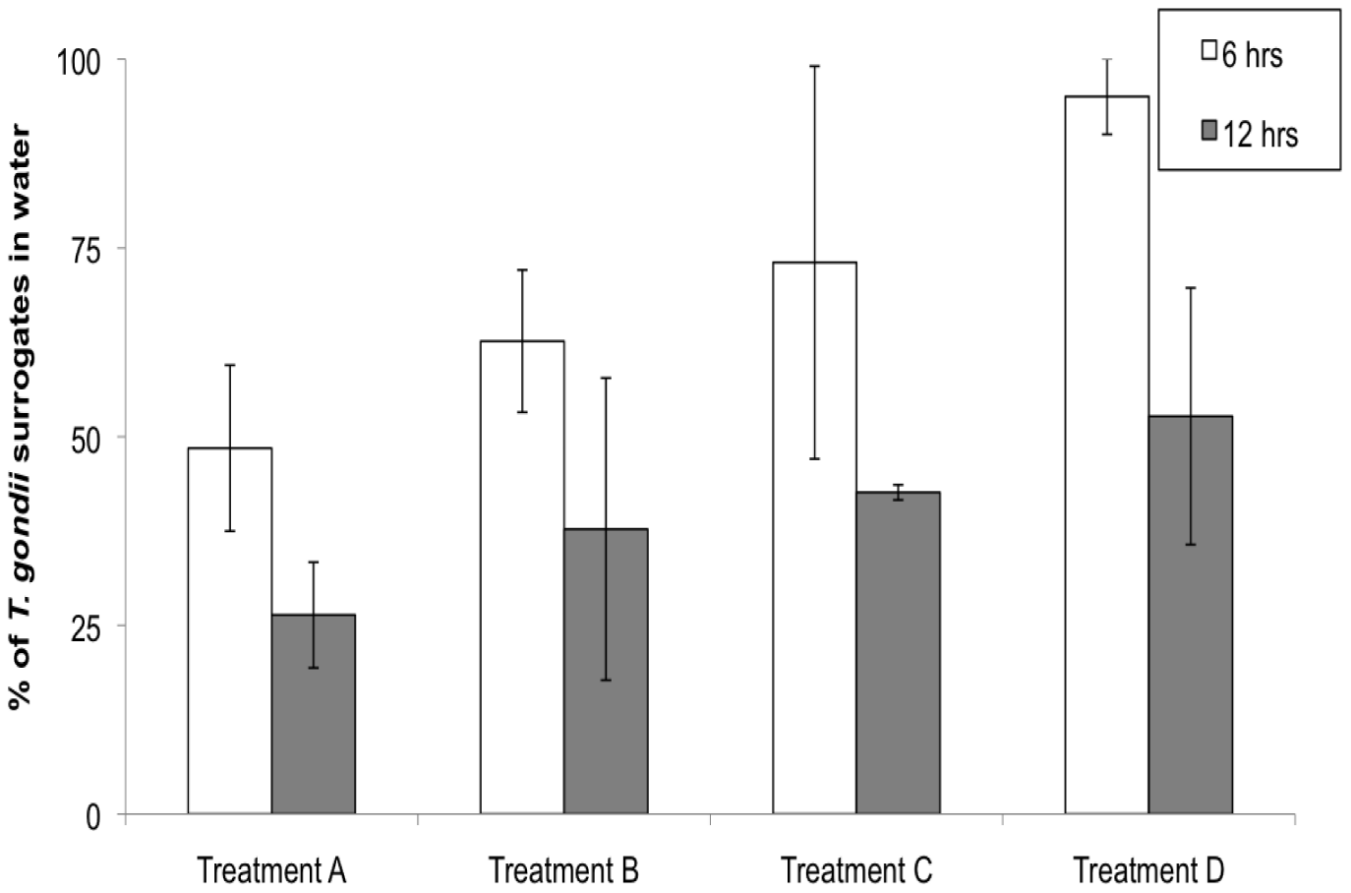
Percentages (mean ± SD) of *T. gondii* oocyst surrogates suspended in the water at samples taken following 6 and 12 hrs during Experiment 2 (N = 9 per treatment, except at t = 6 hrs for treatments C and D where N = 6).

During the experiment (between 6 and 12 hrs), the number of *T. gondii* surrogates suspended in the water decreased significantly in all treatments (Fig. 4, Table 1). Treatment D showed the inherent loss of particles in the water column through the duration of the experiment (Fig. 4).

**Table 1:** Mann-Whitney (or Test U) results shows the significant decrease in the percentage of surrogates of *T. gondii* measured in the water at t = 6 hrs and t = 12 hrs in all treatments.

	Treatment A	Treatment B	Treatment C	Treatment D
*U*	5.00	11	2	1
*p*	<0.05	<0.05	>0.05	>0.05
N	9	9	6	6

**Figure 5:**
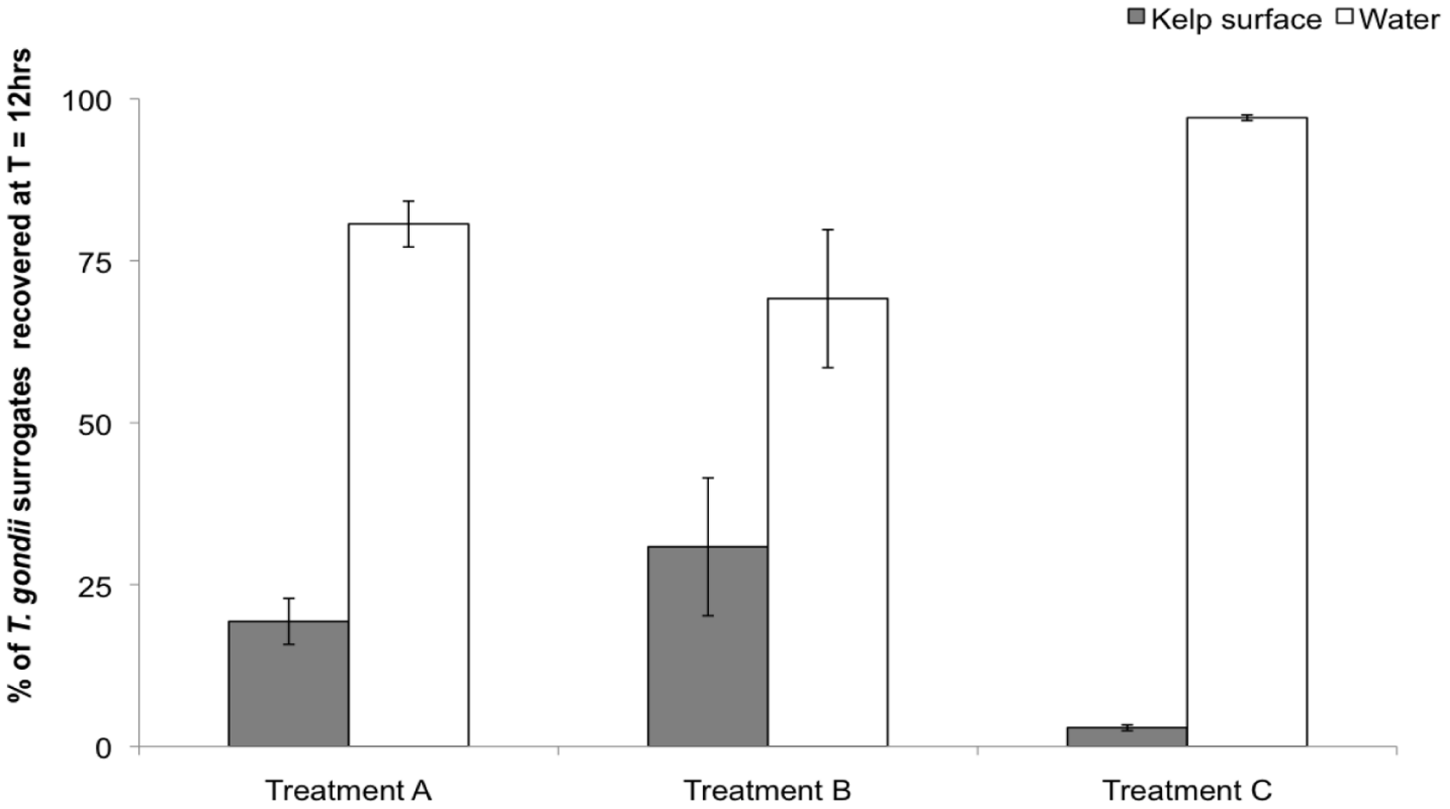
Percentages (mean ± SD) of *T. gondii* surrogates suspended in water and present in kelp scrapings at the termination of Experiment 2 (12 hrs) (N = 9 per treatment).

**Figure 6:**
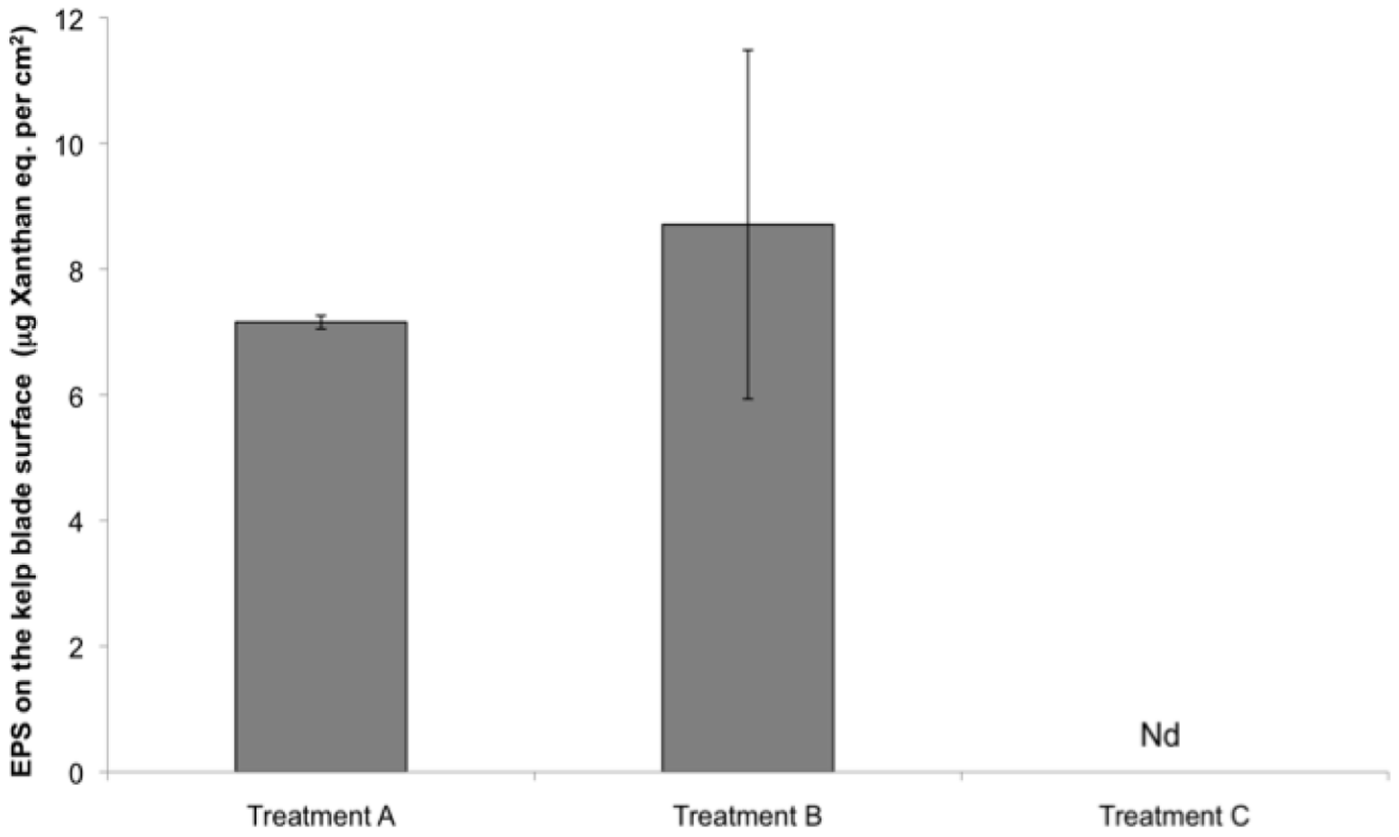
EPS concentrations (mean ± SD) on the kelp blades’ surface at the end of Experiment 2 (t = 12 hrs) (N = 9 per treatment). Nd  =  not detected.

**Figure 7:**
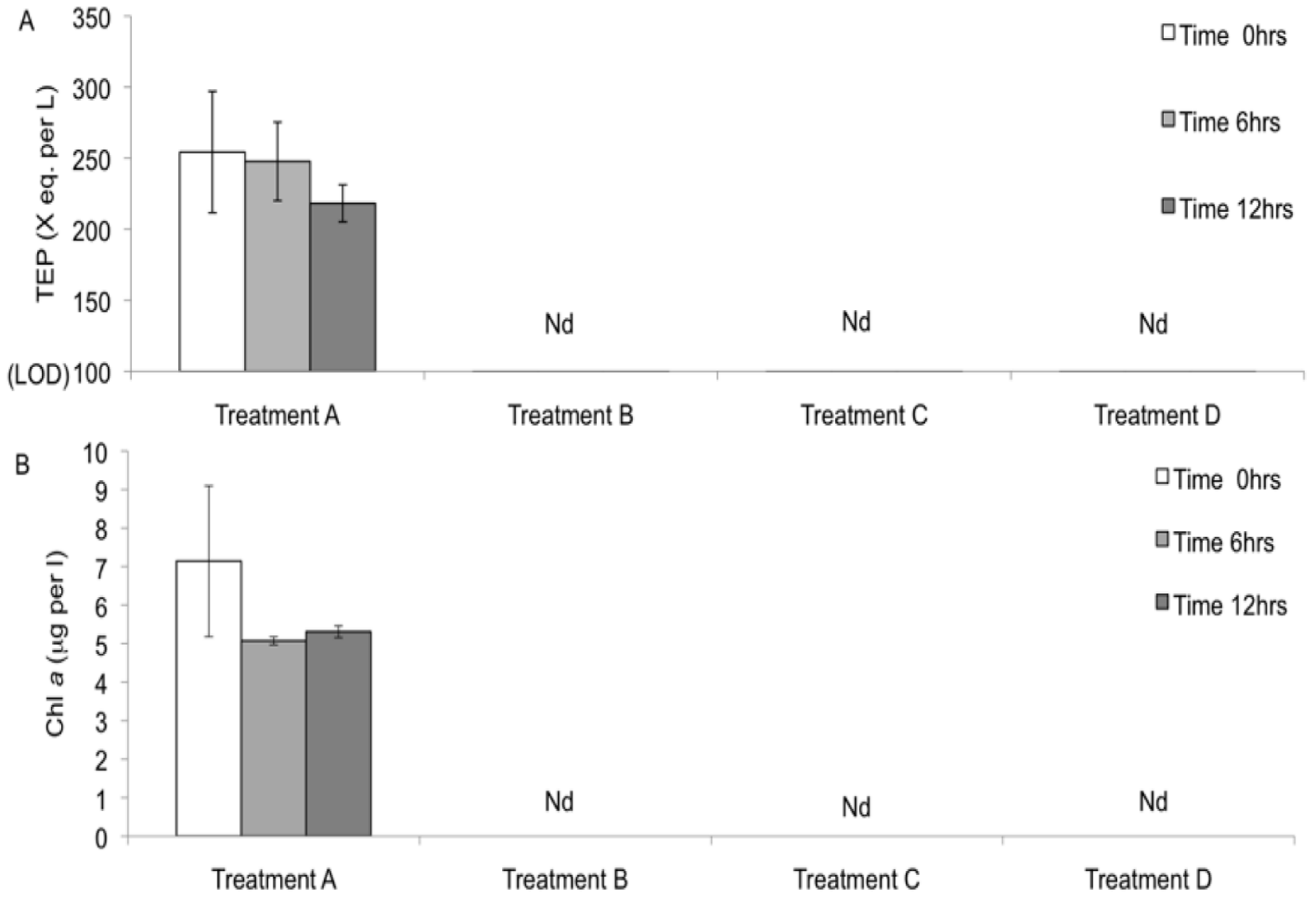
TEP (A) and chlorophyll *a* (B) (mean ± SD) measured in water at t = 0, 6 and 12 hrs in 4 different treatments (N = 9 per treatment) used in Experiment 2. LOD on (A) indicates TEP limit of detection. Nd indicates that TEP and chlorophyll *a* were not detected in treatments B, C, and D.

The percentage of surrogates attached to the kelp blades was calculated relative to the total number of surrogates in the jar (i.e., surrogates in the water + surrogates on the blades) at the end of the experiment (t = 12 hrs). After 12 hrs, the percentage of surrogates associated with kelp blade and suspended in the water significantly differed (Table 2). In the treatment that simulated the kelp forest environment (treatment A), 19% (±3.5) of *T. gondii* oocyst surrogates were found attached to the surfaces of the kelp (Fig. 5), with EPS being detected on the surface of the kelp blade (Fig. 6) and TEP and chlorophyll *a* measured in the water (Fig. 7). The highest percentage of surrogates (31%±10%) was attached to the surface of the kelp blades from treatment B (Fig. 5). In this treatment, kelp blades were covered with EPS (Fig. 6), but TEP and chlorophyll *a* were not detected in the water (Fig. 7). The lowest percentage of surrogates attached to a kelp-like surface was found in the control treatment C that housed the synthetic kelp blades and filtered seawater. In this treatment, there was no measurable TEP in the water nor EPS on the kelp blade surface (Fig. 5, 6 and 7).

**Table 2:** Mann-Whitney results showing significant difference between the percentage of surrogates associate with kelp blades and suspended in the water in all treatments and at the end of the experiment (t =  12 hrs).

	Treatment A	Treatment B	Treatment C
*U*	0	1	0
*p*	<0.05	<0.05	>0.05
N	9	9	9

Kruskal-Wallis results showed that the percentage of surrogates attached to kelp blades significantly differed between treatments with and without EPS covered blades (A and C (*p*<0.05) and treatment B and C (*p*<0.05)), but not between treatments A and B (*p*>0.05), where real kelp blades covered with EPS were used.

**Figure 8:**
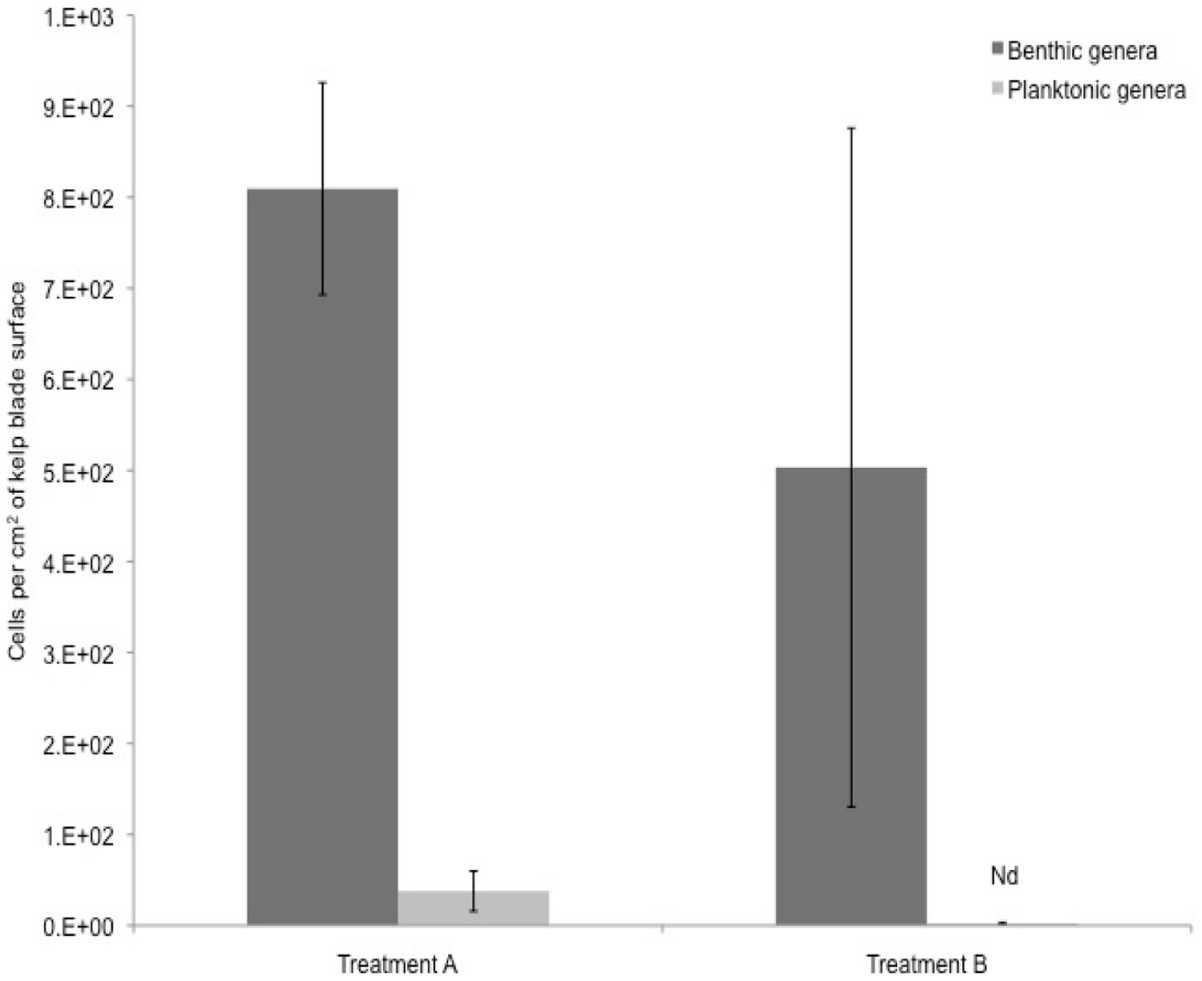
Concentration (mean ± SD) of diatoms on the surface of the kelp blades (N = 3) from Experiment 2 after 12 hrs. Nd  =  not detected.

The concentration of diatoms on kelp blades in samples from treatment A and B, i.e. blades incubated in unfiltered and filtered sea water, respectively, was estimated at 847 (±138) cells per cm^2^ and 505 (±373) cells per cm^2^, respectively. After 12 hrs, in treatment A, 4.2% (±2.1) of the total number of cells associated with the kelp blade surface were diatoms from genera typically observed in the plankton (*Asterionella, Chaetoceros, Cylindrotheca, Ditylum, Eucampia, Hemiaulus, Leptocylindrus, Pseudo-nitzschia, Skeletonema*) (Fig. 8). The remainder included genera of several benthic diatoms. The dominant benthic genera included: *Navicula, Cocconeis, Licmophora and Tabularia*. No diatoms were detected on the synthetic kelp blades housed in 0.2 µm filtered seawater in treatment C.

## Discussion

The goal of the present study was to test whether extracellular polymer substances (EPS), which form the matrix of biofilms colonizing the surfaces of *M. pyrifera* blades, play a role in the transmission of the protozoan parasite *T. gondii* to benthic feeding turban snails. Marine turban snails have been implicated in the exposure of southern sea otters to *T. gondii* [5]. As this parasite has been identified as a significant cause of mortality in endangered southern sea otter populations, it is critically important to understand the transmission mechanisms to otters, so that prevention or management strategies can be developed to reduce likelihood of exposure. Here we show that *T. gondii* surrogates may adhere to the biofilm that colonizes the surfaces of kelp blades, thereby becoming available to turban snails that feed upon organisms associated with this biofilm.

### Turban snail diet and kelp biofilm composition

The surfaces of *Macrocystis pyrifera* blades in the kelp forest canopy were covered with a biofilm composed of benthic diatoms and bacteria embedded in a gel-like matrix. Microscopic observations using the alcian blue stain and quantitative measurements using the colorimetric assay from the 12 hr experiment indicate that the underlying gel-like matrix of this biofilm on the blades is composed of EPS. EPS on kelp blades possibly originates from bacteria and photosynthetic organisms present in the biofilm (i.e., pennate diatoms) and by *M. pyrifera* itself, since EPS production has been linked to photosynthesis and the presence of bacteria [48]–[50]. Other microorganisms (e.g. fungi) may also contribute to the kelp biofilm production system and thereby add to the EPS pool [33]. Locally, we have noted such epizootic populations in our coastal kelp forest communities [20].

**Figure 9:**
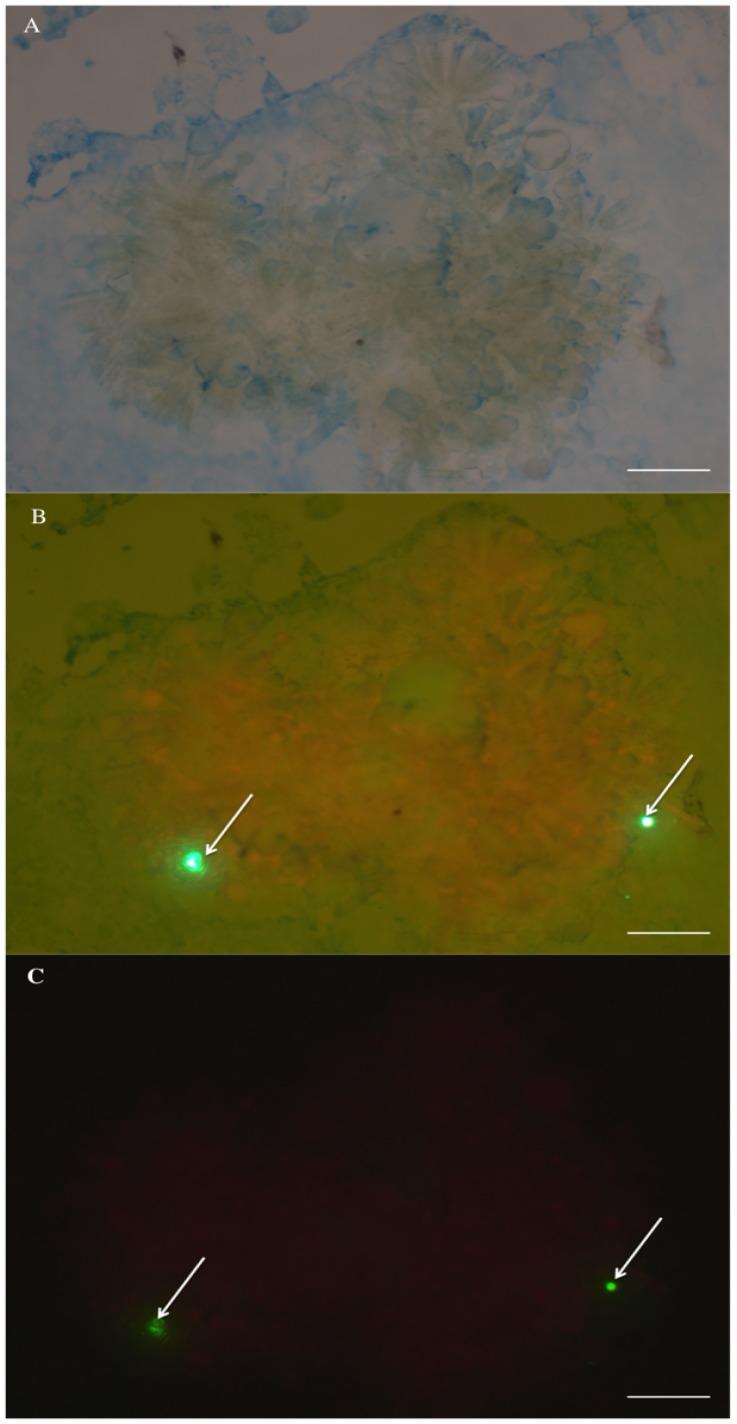
A) Benthic diatoms scraped from the surface of kelp blades analyzed in Experiment 2 showing alcian blue staining of EPS. B) Same image as in A, but observed simultaneously with transmitted light showing both EPS, surrogates of *T. gondii* oocysts (white arrows) and benthic diatoms fluorescing red. C) Same image as in A and B, but observed using 50 W light source and a chlorophyll filter set showing surrogates (white arrows) and chlorophyll (red fluorescence) from benthic diatoms. Scale bars 100 µm.

The high abundance of benthic diatom frustules in fecal pellets of *Chlorostoma* spp. confirms that these subtidal snails feed upon organisms (i.e., pennate diatoms) in kelp blade biofilms. It is likely that the snails are also ingesting EPS, given their mode of food capture: grazing surfaces with a chitinous radula. Sediment dwelling animals also have been shown to ingest EPS attached to sediment particles [51]. The benefits of ingesting EPS may include the fact that EPS are rich in organic carbon and that it may adsorb dissolved organic matter (DOM) providing an important pool of C and N [25]. Results from Experiment 2 indicate that surrogates of *T. gondii* oocysts might be entrapped in the kelp blade biofilm via EPS (Fig. 5 and 9). Therefore, we expect that once oocysts are attached to the kelp blade biofilm they can be consumed as a ‘bycatch’ item by turban snails. Indeed, preliminary findings of a study parallel to ours indicate that turban snails may acquire surrogates of *T. gondii* and *T. gondii* oocysts while they were kept in tanks with kelp blades exposed to surrogates and oocysts [52].

In addition to grazing benthic diatoms that are present within kelp biofilms, *Chlorostoma brunnea* has been suggested to serve as a ‘farmer’ of epizooic fungi that grow on giant kelp surfaces: by wounding the kelp blade with their radula, snails promote fungal infection on the blade. Snails then consume the resultant fungi, with the infection being controlled to maintain an optimal growth rate of the fungus [20]. The presence of such fungal populations on giant kelp has been observed in the field study location, along with their associated microbial communities [20]. Fungi may also produce a large amount of EPS [33] and thus enhance biofilm formation. Although we could not confirm the presence of fungal-like structures in the snail pellets or within the kelp biofilm in our specimens, *T. gondii's* (and other pelagic particles) adhesion to the kelp surfaces and subsequent transmission to snails may be enhanced if fungi are part of the kelp blade biofilm.

### T. gondii surrogate adhesion to kelp blades via EPS

Experiment 2 demonstrated a possible mechanism whereby oocysts of *T. gondii* come to coat the surfaces of kelp blades that are covered with EPS and colonized by benthic diatoms and other microorganisms (Fig. 9). At the end of the 12 hr experiment, an average of 19% and 31% of the total number of surrogates of *T. gondii* oocysts were recovered from kelp blades coated with EPS and their natural associated microorganisms in unfiltered and filtered seawater treatments, respectively. These results suggest a novel mechanism by which contaminated runoff entering the coastal ocean can deliver *T. gondii* oocysts to downstream kelp beds, with some of the oocysts adhering to kelp surfaces covered with EPS.

The mechanism of parasite adhesion to surfaces of kelp covered by EPS is likely related to hydrophobic and electrostatic attractive forces, which are influenced by the composition of the oocyst outer wall [53]. The *T. gondii* oocyst cell wall consists of a matrix of polymeric substances, mostly proteins including cysteine-rich proteins among others [54]. The cysteine-rich proteins of *T. gondii* oocyst are related to those of the walls of another coccidian parasite, *Cryptosporidium* oocyst, [55]. Interestingly, the adhesion of *Cryptosporidium* oocysts to biofilms on man-made surfaces has been verified and the roughness of the biofilm has been strongly correlated with oocyst retention [56]–[58]. *Giardia* cysts, which are also covered with a polymeric matrix, have also been documented to attach to biofilms [59]. Thus, our discovery that surrogates of *T. gondii* oocysts attach to biofilm that covers the surfaces of kelp is consistent with previous findings that environmentally resistant parasites can adhere to surfaces covered with sticky biofilms.

Additional evidence for a mechanism promoting adhesion of suspended particles to surfaces covered with EPS was provided by our observation that planktonic diatoms were present on surfaces of kelp blades. Planktonic diatoms, likely present in the unfiltered seawater at the start of our experiment, presumably were the source of the diatoms that we observed attached to the kelp surfaces. Thus, our study provides evidence of adhesion of planktonic diatoms and suspended particles such as *T. gondii* oocysts to surfaces covered with an EPS biofilm.

The mechanism whereby EPS serves as an adhesive may have implications in other fields of research. Biofilms occur in a variety of environments (surfaces of rocks, plants, sediments, ship hulls or wastewater treatment plants). Zoonotic pathogens other than *T. gondii* that can infect both humans and marine animals have been detected in the coastal environment. For example, (oo)cysts of *Cryptosporidium* and *Giardia* as well as enteric bacteria have been documented in the coastal ocean and found to infect marine fauna [60]–[62]. Although the route of infection of marine wildlife by these parasites is still unclear, it is possible that these pathogens also may become associated, via EPS, with biofilms that coat natural or man-made surfaces in the ocean. The novelty provided by these results is a proposed mechanism by which land-derived pathogens in contaminated runoff are transferred from the water column to a benthic environment, thus facilitating the exposure of benthic-feeding marine fauna to *T. gondii* and other pathogens.

It is unclear whether the presence of TEP in the seawater influenced the adherence of oocyst surrogates to kelp blades that had EPS coatings. TEP can be regarded as part of the particle pool of EPS [63], with both TEP and EPS containing acidic polysaccharides and possessing adhesive properties [31], [64]. In a kelp forest environment, TEP may be detected in the water ([65], pers. obs.) and may serve as an agent to deliver oocysts to EPS-covered kelp blades. In our experiment, only a small difference was detected between the mean proportions of surrogates recovered from kelp in treatments with and without TEP (Fig 5., treatments A and B), with more surrogates being recovered from kelp without TEP.

The hypothesis that TEP can influence the adhesion of oocysts to surfaces should be further investigated. Biofilms of TEP origin develop quickly on surfaces submerged in filtered seawater [35]. Results from treatments C and D suggest that TEP may deliver surrogates to surfaces (Fig. 4): even though we removed all TEP producers by filtering seawater (at 0.2 µm), TEP precursors in the colloidal form may have been present and could have formed TEP over the 12 hr incubation period, with concentrations possibly being below our limit of detection. Surrogates could therefore have become associated with these TEP and thereby delivered to the surfaces of the synthetic kelp blades and surfaces within the experimental containers. However, the inherent ‘loss’ of surrogates through experimental steps is also observed in experiments that use these particles in ultra purified water and recovery of all surrogates rarely occurs. The influence of TEP in oocyst delivery to kelp blades should be further investigated, perhaps in a similar experiment using a method that would detect TEP at lower concentrations.

## Conclusions

Our findings suggest a novel route of exposure of sea otters to the protozoan parasite *T. gondii*. Although the estimated number of *T. gondii* oocysts that are transported to kelp forests is unknown, these experimental results provide a mechanism to explain the transmission of *T. gondii* oocysts to sea otters. As *T. gondii* oocysts are deposited in the coastal ocean via contaminated runoff, we propose that a proportion of them attach to the sticky EPS biofilms on the kelp blades, with the surface communities composed in part of benthic diatoms and bacteria. Snails, which feed by scraping these benthic diatoms from the surface of kelp blade using their radula, would ingest *T. gondii* oocysts as ‘bycatch’, explaining why sea otters that specialize on consuming subtidal snails are more likely to be exposed to this parasite. The route of infection of other pathogens to marine wildlife may also occur via an EPS-adhesion mechanism such as that described here. Insight into EPS-mediated pathogen transmission may also have significant implications for human public health, due to consumption of marine animals that feed on EPS-coated substances [66]. This study, therefore, suggests a new transmission route for delivering microscopic-sized pelagic pathogens to higher trophic level predators in marine ecosystems.

## References

[1] Tenter AM, Heckeroth AR, Weiss LM (2000) *Toxoplasma gondii*: from animals to humans. Int. J. Parasitol 30: 1217–1258. 10.1016/s0020-7519(00)00124-7 PMC3109627 11113252

[2] Miller MA (2008) Tissue cyst-forming coccidia of marine mammals. In: Fowler M, Miller R, editors. Zoo and wild animal medicine. Sounders Elsevier. pp. 319–340.

[3] Kreuder C, Miller MA, Jessup DA, Lowenstine LJ, Harris MD, et al (2003) Patterns of mortality in southern sea otters (*Enhydra lutris nereis*) from 1998–2001. J Wildlife Dis 39: 495–509. 10.7589/0090-3558-39.3.495 14567210

[4] Miller MA, Gardner IA, Kreuder C, Paradies DM, Worcester KR, et al (2002) Coastal freshwater runoff is a risk factor for *Toxoplasma gondii* infection of southern sea otters (*Enhydra lutris nereis*). Int J Parasitol 32: 997–1006. 12076629 10.1016/s0020-7519(02)00069-3

[5] Johnson CK, Tinker MT, Estes JA, Conrad PA, Staedler M, et al (2009) Prey choice and habitat use drive sea otter pathogen exposure in a resource-limited coastal system. Proc Nat Acad Sci USA 106: 2242–2247. 19164513 10.1073/pnas.0806449106 PMC2650139

[6] Conrad PA, Miller MA, Kreuder C, James ER, Mazet J, et al (2005) Transmission of *Toxoplasma*: clues from the study of sea otters as sentinels of *Toxoplasma gondii* flow into the marine environment. Int J Parasitol 35: 1155–1168. 16157341 10.1016/j.ijpara.2005.07.002

[7] Hutchison WM, Dunachie JF, Siim JC, Work K (1969) Life cycle of *Toxoplasma gondii* . Brit Med J 4: 806. 10.1136/bmj.4.5686.806-b PMC1630290 5359949

[8] Dubey AJP, Andrews C, Lind P, Kwok O, Thulliez P, et al (1996) Antibody responses measured by various serologic tests in pigs orally inoculated with low numbers of *Toxoplasma gondii* oocysts. Am J Vet Res 57: 1733–1737. 8950427

[9] Dubey AJP (1996) Pathogenicity and Infectivity of *Toxoplasma gondii* oocysts for rats. J Parasitol 82: 951–956. 8973405

[10] Dubey AJP, Lunney JK, Shen SK, Kwok OCH, Ashford DA, et al (1996) Infectivity of low numbers of *Toxoplasma gondii* oocysts to pigs. J Parasitol 82 (30): 438–443. 8636849

[11] Shapiro K, Conrad PA, Mazet JAK, Wallender WW, Miller WA, et al (2010) Effect of estuarine wetland degradation on transport of *Toxoplasma gondii* surrogates from land to sea. Appl Environ Microbiol 76: 6821–6828. 20802072 10.1128/AEM.01435-10 PMC2953021

[12] Lindsay DS, Dubey JP (2009) Long-term survival of *Toxoplasma gondii* sporulated oocysts in seawater. J Parasitol 95: 1019–1020. 20050010 10.1645/GE-1919.1

[13] Shapiro K, Silver MW, Largier JL, Conrad PA, Mazet JAK (2012) Association of *Toxoplasma gondii* oocysts with fresh, estuarine, and marine macroaggregates. Limnol Ocean 57: 449–456.

[14] Lindsay DS, Phelps K, Smith S, Flick G, Dubey J (2001) Removal of *Toxoplasma gondii* oocysts from seawater by easter oysters (*Crassostrea virginica*). J Euk Microbiol: 197S–198S. 10.1111/j.1550-7408.2001.tb00517.x 11906061

[15] Arkush KD, Miller MA, Leutenegger CM, Gardner IA, Packham AE, et al (2003) Molecular and bioassay-based detection of *Toxoplasma gondii* oocyst uptake by mussels (*Mytilus galloprovincialis*). Int J Parasitol 33: 1087–1097. 13129531 10.1016/s0020-7519(03)00181-4

[16] Miller MA, Miller WA, Conrad PA, James ER, Melli AC, et al (2008) Type X *Toxoplasma gondii* in a wild mussel and terrestrial carnivores from coastal California: new linkages between terrestrial mammals, runoff and toxoplasmosis of sea otters. Int J Parasitol 38: 1319–1328. 18452923 10.1016/j.ijpara.2008.02.005

[17] Massie GN, Ware MW, Villegas EN, Black MW (2010) Uptake and transmission of *Toxoplasma gondii* oocysts by migratory, filter-feeding fish. Vet Parasitol 169: 296–303. 20097009 10.1016/j.vetpar.2010.01.002

[18] Watanabe J (1984) Food preference, food quality and diets gastropods (Trochidae: *Tegula*) of three herbivorous in a temperate kelp forest habitat. Oecol 62: 47–52. 10.1007/BF00377371 28310736

[19] Mazzella L, Russo GF (1989) Grazing effect of two *Gibbula* species (Mollusca, Archaeogastropoda) on the epiphytic community of *Posidonia oceanica* leaves. Aquatic Bot 35: 357–373.

[20] McMillan S (2010) Trophic interactions among *Chlorostoma brunnea*, *Macrocystis pyrifera*, and fungi. San Jose State University. Available: http://scholarworks.sjsu.edu/etd_theses/3777.

[21] Stoodley P, Sauer K, Davies DG, Costerton JW (2002) Biofilms as complex differentiated communities. Annu Rev Microbiol 56: 187–209. 12142477 10.1146/annurev.micro.56.012302.160705

[22] Wotton RS (2005) The essential role of exopolymers (EPS) in aquatic systems. In: Barnes H. editor. Oceanography and Marine Biology. CRC Press. pp. 57–94.

[23] Pal A, Paul AK (2008) Microbial extracellular polymeric substances: central elements in heavy metal bioremediation. Indian J Appl Microbiol 48: 49–64. 10.1007/s12088-008-0006-5 PMC3450203 23100700

[24] Decho A (2009) In situ Imaging and Characterizing the Matrix of Extracellular Polymeric Substances (EPS) of Biofilms. Microsc Microanal 15: 822–823.

[25] Decho AW (2000) Microbial biofilms in intertidal systems: an overview. Cont Shelf Res 20: 1257–1273.

[26] Decho AW, Lopez GR (1993) Exopolymer microenvironments of microbial flora: Multiple and interactive effects on trophic relationships. Limnol Ocean 38: 1633–1645.

[27] Wetherbee R, Lind JL, Burke J, Quatrano RS (1998) Minireview-the First Kiss: Establishment and Control of Initial Adhesion By Raphid Diatoms. J Phycol 34: 9–15.

[28] Krembs C, Eicken H, Junge K, Deming J (2002) High concentrations of exopolymeric substances in Arctic winter sea ice: implications for the polar ocean carbon cycle and cryoprotection of diatoms. Deep Sea Res Pt I 49: 2163–2181.

[29] Simental J, Sanchez-Saavedra M, Flores-Acevedo N (2004) Growth and survival of juvenile red abalone (*Haliotis rufescens*) fed with macroalgae enriched with a benthic diatom film. J Shellfish Res 23: 995–999.

[30] Del Pilar Sanchez-Saavedra M (2008) Removal of epiphytes of the kelp *Macrocystis pyrifera* (L.) Agardh using different biocides. Hidrobiologica 18: 99–104.

[31] Sutherland I (2001) Biofilm exopolysaccharides: a strong and sticky framework. Microbiol 147: 3–9. 10.1099/00221287-147-1-3 11160795

[32] Hoagland KD, Rosowski JR, Gretz MR, Roemer SC (1993) Diatom extracellular polymeric substances: function, fine structure, chemistry and physiology. J Phycol: 537–566.

[33] Selbmann L, Stingele F, Petruccioli M (2003) Exopolysaccharide production by filamentous fungi: the example of *Botryosphaeria rhodina* . Antonie van Leeuwenhoek 84: 135–145. 14533717 10.1023/a:1025421401536

[34] Passow U (2002) Transparent exopolymer particles (TEP) in aquatic environments. Prog Oceanogr 55: 287–333.

[35] Berman T, Passow U (2007) Transparent Exopolymer Particles (TEP): an overlooked factor in the process of biofilm formation in aquatic environments. Nature Preceedings: 1–13.Available: doi:10.1038/npre.2007.1182.1. Accessed 10 November 2012

[36] Bar-Zeev E, Berman-Frank I, Girshevitz O, Berman T (2012) Revised paradigm of aquatic biofilm formation facilitated by microgel transparent exopolymer particles. Proc Nat Acad Sci USA 109: 9119–9124. 22615362 10.1073/pnas.1203708109 PMC3384133

[37] Molino PJ, Wetherbee R (2008) The biology of biofouling diatoms and their role in the development of microbial slimes. Biofouling 24: 365–379. 18604655 10.1080/08927010802254583

[38] Angles ML, Chandy JP, Cox PT, Fisher IH, Warnecke MR (2007) Implications of biofilm-associated waterborne *Cryptosporidium* oocysts for the water industry. Trends Parasitol 23: 352–356. 17574922 10.1016/j.pt.2007.06.001

[39] VanWormer E, Fritz H, Shapiro K, Mazeta JAK, Conrad PA (2013) Molecules to modeling: *Toxoplasma gondii* oocysts at the human-animal-environment interface. Comp Immunol, Microb 36: : 217– 231. 10.1016/j.cimid.2012.10.006 PMC3779781 23218130

[40] Shapiro K, Largier J, Mazet JAK, Bernt W, Eli JR, et al (2009) Surface properties of *Toxoplasma gondii* oocysts and surrogates micrsopheres. Appl Environ Microbiol 75 (4): 1185–1191. 19060174 10.1128/AEM.02109-08 PMC2643564

[41] Passow U, Alldredge A (1995) A dye-binding assay for the spectrophotometric measurement of transparent exopolymer particles (TEP). Limnol Ocean 40: 1326–1335.

[42] Shapiro K, Mazet JAK, Schriewer A, Wuertz S, Fritz H, et al (2010) Detection of *Toxoplasma gondii* oocysts and surrogate microspheres in water using ultrafiltration and capsule filtration. Water Res 44: 893–903. 19836820 10.1016/j.watres.2009.09.061

[43] Arar EJ, Collins GB (1997) *In vitro* determination of chlorophyll *a* and pheophytin *a* in marine freshwater algae by fluorescence. In: Dufour A. editor. Methods for the determination of chemical substances in marine and estuarine environmental samples. U.S. Environmental Protection Agency, Cincinnati, Ohio 45268. Method 445.0.

[44] Krembs C, Eicken H, Deming JW (2011) Exopolymer alteration of physical properties of sea ice and implications for ice habitability and biogeochemistry in a warmer Arctic. Proc Nat Acad Sci USA 108: 3653–3658. 21368216 10.1073/pnas.1100701108 PMC3048104

[45] Vandevivere P, Kirchman DL (1993) Attachment Stimulates Exopolysaccharide Synthesis by a Bacterium. Appl Environ Microbiol 59: 3280–3286. 16349064 10.1128/aem.59.10.3280-3286.1993 PMC182449

[46] Engel A, Passow U (2001) Carbon and nitrogen content of transparent exopolymer particles (TEP) in relation to their Alcian Blue adsorption. Mar Ecol Prog Ser 219: 1–10.

[47] Utermöhl H (1958) Zur Vervollkommnung der quantitativen Phytoplanktonmethodik. Mitt Int Ver Limnol 9..

[48] Rochelle-Newall EJ, Mari X, Pringault O (2010) Sticking properties of transparent exopolymeric particles (TEP) during aging and biodegradation. J Plankton 32: 1433–1442.

[49] Harimawan A, Rajasekar A, Ting YP (2011) Bacteria attachment to surfaces-AFM force spectroscopy and physicochemical analyses. J Colloid Interf Sci 364: 213–218. 10.1016/j.jcis.2011.08.021 21889162

[50] Smith DJ, Underwood GJC (2001) The production of extracellular carbohydrates by estuarine benthic diatoms: the effects of growth phase and light and dark treatment. J Phycol 36: 321–333.

[51] Hoskins D, Stancyk S, Decho A (2003) Utilization of algal and bacterial extracellular polymeric secretions (EPS) by the deposit-feeding brittlestar *Amphipholis gracillima* (Echinodermata). Mar Ecol Prog Ser 247: 93–101.

[52] Krusor C (2012) Acquisition, concentration, and retention of *Toxoplasma gondii* oocysts from seawater by marine snails. University of California, Davis. Proquest, UMI Dissertations Publishing, 2012. Interm No.1534893.

[53] Dumètre A, Aubert D, Puech PH, Hohweyer J, Azas N, et al (2012) Interaction forces drive the environmental transmission of pathogenic protozoa. Appl Environ Microbiol 78: 905–912. 22156429 10.1128/AEM.06488-11 PMC3273037

[54] Fritz HM, Bowyer PW, Bogyo M, Conrad PA, Boothroyd JC (2012) Proteomic analysis of fractionated *Toxoplasma* oocysts reveals clues to their environmental resistance. Plosone 7: e29955. 10.1371/journal.pone.0029955 PMC3261165 22279555

[55] Possenti A, Cherchi S, Bertuccini L, Pozio E, Dubey JP, et al (2010) Molecular characterisation of a novel family of cysteine-rich proteins of *Toxoplasma gondii* and ultrastructural evidence of oocyst wall localization. Int J Parasitol 40: 1639–1649. 20708619 10.1016/j.ijpara.2010.06.009

[56] Searcy KE, Packman AI, Atwill ER, Harter T (2006) Capture and retention of *Cryptosporidium parvum* oocysts by *Pseudomonas aeruginosa* biofilms. Appl Environ Microbiol 72: 6242–6247. 16957251 10.1128/AEM.00344-06 PMC1563676

[57] Liu Y, Janjaroen D, Kuhlenschmidt MS, Kuhlenschmidt TB, Nguyen TH (2009) Deposition of *Cryptosporidium parvum* oocysts on natural organic matter surfaces: microscopic evidence for secondary minimum deposition in a radial stagnation point flow cell. Langmuir 25: 1594–1605. 19133757 10.1021/la803202h

[58] DiCesare EAW, Hargreaves BR, Jellison KL (2012) Biofilm roughness determines *Cryptosporidium parvum* retention in environmental biofilms. Appl Environ Microbiol 78: 4187–4193. 22492449 10.1128/AEM.08026-11 PMC3370560

[59] Helmi K, Skraber S, Gantzer C, Willame R, Hoffmann L, et al (2008) Interactions of *Cryptosporidium parvum*, *Giardia lamblia*, vaccinal poliovirus type 1, and bacteriophages phiX174 and MS2 with a drinking water biofilm and a wastewater biofilm. Appl Environmental Microbiol 74: 2079–2088. 10.1128/AEM.02495-07 PMC2292593 18281435

[60] Oates SC, Miller MA, Byrne BA, Chouicha N, Hardin D, et al (2012) Epidemiology and potential land-sea transfer of enteric bacteria from terrestrial to marine species in the Monterey Bay region of California. J Wildlife Dis 48: 654–668. 10.7589/0090-3558-48.3.654 22740531

[61] Shapiro K, Miller M, Mazet J (2012) Temporal association between land-based runoff event and California sea otter (*Enhydra lutris nereis*) protozoal mortalities. J Wildlife Dis 48: 394–404. 10.7589/0090-3558-48.2.394 22493114

[62] Fayer R, Dubey JP, Lindsay DS (2004) Zoonotic protozoa: from land to sea. Trends Parasitol 20: 531–536. 15471705 10.1016/j.pt.2004.08.008

[63] Thornton DCO (2002) Diatom aggregation in the sea: mechanisms and ecological implications. Eur J Phycol 37: 149–161.

[64] Engel A (2002) Direct relationship between CO_2_ uptake and transparent exopolymer particles production in natural phytoplankton. J Plankton Res 24: 49–53.

[65] Ramaiah N, Yoshikawa T, Furuya K (2001) Temporal variations in transparent exopolymer particles (TEP) associated with a diatom spring bloom in a subartic area in Japan. Mar Ecol Prog Ser 212: 79–88.

[66] Jones JL, Dargelas V, Roberts J, Press C, Remington JS, et al (2009) Risk factors for *Toxoplasma gondii* infection in the United States. Clin Infect Dis 49 (6): 878–884. 19663709 10.1086/605433

